# CXCR2 in Acute Lung Injury

**DOI:** 10.1155/2012/740987

**Published:** 2012-06-06

**Authors:** F. M. Konrad, J. Reutershan

**Affiliations:** Department of Anesthesiology and Intensive Care Medicine, University Hospital of Tübingen, 72076 Tübingen, Germany

## Abstract

In pulmonary inflammation, recruitment of circulating polymorphonuclear leukocytes is essential for host defense and initiates the following specific immune response. One pathological hallmark of acute lung injury and acute respiratory distress syndrome is the uncontrolled transmigration of neutrophils into the lung interstitium and alveolar space. Thereby, the extravasation of leukocytes from the vascular system into the tissue is induced by chemokines that are released from the site of inflammation. The most relevant chemokine receptors of neutrophils are CXC chemokine receptor (CXCR) 1 and CXCR2. CXCR2 is of particular interest since several studies implicate a pivotal role of this receptor in development and promotion of numerous inflammatory disorders. CXCR2 gets activated by ELR^+^ chemokines, including MIP-2, KC (rodents) and IL-8 (human). Since multiple ELR^+^ CXC chemokines act on both receptors—CXCR1 and CXCR2—a pharmacologic agent blocking both receptors seems to be advantageous. So far, several CXCR1/2 antagonists have been developed and have been tested successfully in experimental studies. A newly designed CXCR1 and CXCR2 antagonist can be orally administered and was for the first time found efficient in humans. This review highlights the role of CXCR2 in acute lung injury and discusses its potential as a therapeutic target.

## 1. Introduction

As an essential component of the unspecific immune defense, leukocytes migrate from the blood into inflammatory tissue. Uncontrolled, excessive infiltration of leukocytes into the tissue leads to a destruction of organ structure and is a main characteristic of acute and chronic inflammatory disorders like atherosclerosis, reperfusion injury or acute lung injury (ALI) [1].

ALI and its more severe form acute respiratory distress syndrome (ARDS) are still life-threatening syndromes primarily seen on intensive care units with a mortality rate of 40% in approximately 200,000 adult patients per year in the USA [2]. There, the incidence of ALI was presented as high as 86.2 per 100,000 person-years [2]. So far, outcome can not be improved by pharmacologic treatments (e.g., inhaled NO, surfactant, glucocorticoids, antioxidants) [3]. Only ventilation with lower tidal volumes has shown some benefit [4].

In pulmonary inflammation, recruitment of circulating polymorphonuclear leukocytes (PMNs) is essential for defense and reducing the bacterial burden in the alveolar space—and initiates the following specific immune response. If PMN migration into the lung is impaired, the immune response is severely disturbed [5]. PMN migration into the lung proceeds by several steps upon an inflammatory stimulus: first, accumulation of PMNs in the capillaries, then transendothelial migration into the lung interstitium and finally, transepithelial migration into the alveolar space. Each migration step is regulated differently [6]. Uncontrolled transmigration of PMNs into the interstitium of the lung and alveolar space is a pathologic hallmark of ALI/ARDS [7]. Experimental studies proved that modulation of PMN trafficking improves tissue destruction and the outcome of ALI [6, 8], whereas persisting neutrophilia is associated with poor outcome [9].

The extravasation of leukocytes is controlled by chemokines, which are released at the site of inflammation and induce chemotaxis. Chemokines are small, soluble peptides and interact with cells through specific chemokine receptors. Besides chemotaxis, chemokines can activate integrins that mediate leukocyte adherence on endothelial cells. The existence of the multiplicity of chemokines and their specific receptors enable selective trafficking of different immune cells under normal and inflammatory conditions [10–12]. So far, the existence of more than 40 members of chemokines and 19 different chemokine receptors has been demonstrated [13], and most chemokine receptors have multiple chemokine ligands.

Neutrophils are an essential component of the innate immune system and are the first group of cells that migrate to sites of infection. CXC chemokine receptor (CXCR) 1 and CXCR2 are the major chemokine receptors of neutrophils [14–16]. CXCR2 is of particular interest since several studies implicate a pivotal role of this receptor in development and promotion of tumor progression and numerous inflammatory disorders [17–24]. Acute and chronic inflammatory conditions involving CXCR2 contain ischemia/reperfusion injury, chronic obstructive pulmonary disease and fibrosis [25, 26]. In CXCR2−/− mice, neutrophil migration to sites of inflammation is severely disturbed [27]. In experimental approaches, CXCR2 antagonism is able to attenuate tissue damage and disease progress; for example in radiation-induced alveolitis, sepsis, peritonitis and arthritis [28–31]. Modulation of the function of CXCR2 is therefore considered as a possible therapeutic strategy in the treatment of inflammatory conditions in humans [32].

## 2. CXCR2 Structure and Its Ligands

Like most chemokine receptors, CXCR2 is a G protein-coupled receptor assembled by seven transmembrane domains and connected with heterotrimeric G proteins. CXCR2 (and CXCR1) is expressed by granulocytes, especially neutrophils, but also by eosinophils, mast cells, T lymphocytes and basophils [13, 39]. CXCR2 is also found on endothelial cells where it also contributes to chemotaxis [48]. In addition, CXCR2 is expressed in liver, kidney, and in cells of the central nervous system.

CXCR1 and CXCR2 share 78% of their amino acid sequence and get activated by ELR^+^ chemokines (glutamic acid-leucine-arginine containing). Generally, ELR^+^ chemokines have angiogenic activity [49] and therefore play a pivotal role in the exudative and fibroproliferative phases of ARDS [50]. ELR^+^ chemokines are potent neutrophil chemoattractants and activators and, when administered exogenously, induce neutrophil mobilization from the bone marrow into the circulation [51–54].

CXCR2 is the receptor for seven structurally related ELR^+^ chemokines: CXCL1, CXCL2, CXCL3, CXCL6, and CXCL8 (see Table 1 for details). CXCL8 and, to a lower degree CXCL6, are agonists for CXCR1 and CXCR2; whereas the other ELR^+^ chemokines selectively bind to and activate CXCR2 [32, 55].

The murine CXCL1 and CXCL2/3, functional homologues of human growth-related oncogenes (GRO*α*, -*β*, and -*γ*), and CXCL 8, have been identified as the primary chemokine receptor system mediating neutrophil recruitment in various models of acute and chronic inflammation [26, 48, 56].

## 3. CXCR in Rodents

For a long time, rodents were believed to express only a functional CXCR2 homologue, although an early study demonstrated the presence of both CXCR subtypes in mice by southern blot [57]. In the last years, however, increasing evidence for existence and—more important—biological activity of CXCR1 in mice has been demonstrated. One group identified the homologues receptor to CXCR1 in mice on chromosome 1 that is expressed on several cell types like neutrophils, and in the kidney, liver, and spleen [58]. Since this receptor failed to be activated by known human and murine CXC chemokines [58], it remains questionable whether this receptor is functionally analog to the human CXCR1. Another group found a possible CXCR1-gene in mice genome that is 77% identical to rat gene and 58% identical to human gene for CXCR1 [59]. In an LPS-induced murine model, this potential CXCR1-gene was up-regulated in PMNs that migrated into the lung [59], but there are further studies on functional characteristics necessary to confirm this possible CXCR1-gene. Recent data demonstrated expression of CXCR1 in mice with the corresponding ligand CXCL6/GCP-2, both receptor and ligand associated with murine collagen-induced arthritis [60]. There is still additional research required to answer the question whether rodents express CXCR1 and if so, how it interacts with CXCR2.

## 4. CXCR2 in Acute Lung Injury

Blocking CXCR2 inhibits the release of neutrophils from the bone marrow; consequently, exogenous administration of CXCL1 and CXCL2 increases the release [1, 61–64]. The emigration of neutrophils from the bone marrow is disturbed in mice lacking CXCR2 [62], causing a similar disease pattern as the congenital disorder myelokathexis, which is characterized by chronic neutropenia due to defective emigration of PMNs from the bone marrow.

In humans, elevated levels of multiple ELR^+^ CXC chemokines were found in the bronchoalveolar lavage fluid of patients after ischemia-reperfusion injury [18]. In rats, lung expression of CXCL1, CXCL2/3, and their receptor CXCR2 were accompanied by lung neutrophil infiltration and injury. Inhibition of CXCR2 or CXCR2 ligands led to a marked reduction of migrated neutrophils into the lung and graft injury [18].

In CXCR2−/− mice, PMN migration into the lung was significantly reduced in LPS-induced ALI [48]. This effect couldn't only be ascribed to the lack of CXCR2 on PMNs as demonstrated in chimeric mice. Mice with CXCR2 only on hematopoietic cells—like neutrophils—still showed reduced PMN migration into the lung, but at a higher amount compared to CXCR−/− mice. Mice with CXCR2 only on non-hematopoietic cells—like endothelial or epithelial cells—also demonstrated a defect in PMN recruitment, but again, not as much as in CXCR−/− mice. Therefore, CXCR2 on hematopoietic and non-hematopoietic cells seems to play a pivotal role in PMN recruitment. In the same approach, microvascular permeability was measured by Evans blue extravasation, and also required CXCR2 on non-hematopoietic cells.

In hyperoxia-induced ALI, CXCR2−/− mice showed significantly reduced neutrophil sequestration and lung injury and had a survival advantage compared with CXCR2+/+ mice [65]. In a model of ventilator-induced lung injury, inhibition of CXCR2 led to a marked reduction in neutrophil sequestration and lung injury [17]. In bleomycin-induced lung injury, treatment with a CXCR2 receptor antagonist attenuated airway neutrophils but increased neutrophils in lung parenchyma [26]. CXCR2-blocked animals showed improved lung pathology and diminished collagen deposition. Cigarette smoke exposure induced acute inflammation in the lungs of mice [38]. Blocking CXCR2 with SCH-N led to a reduced number of neutrophils in the bronchoalveolar lavage, decreased tissue neutrophils, and attenuated perivascular inflammation. In a pig model of aspiration of bacterial-laden gastric contents, blocking the CXCR2 axis reduced pulmonary neutrophilia and pathology, while maintaining pulmonary bacterial clearance [41].

In one clinical study on CXCR2, 18 healthy volunteers were exposed to a moderate dose of ozone (250 ppb, 3 h of intermittent moderate exercise). Ozone induces a transient increase of 20% in sputum neutrophils [46]. Pharmacological blocking of CXCR2 with the antagonist SCH527123 resulted in significantly lower sputum neutrophil counts.

## 5. Pharmacological CXCR2 Blocking Strategies

Since multiple ELR^+^ CXC chemokines act on both receptors—CXCR1 and CXCR2—, a pharmacologic agent blocking both receptors seems to be desirable [39]; even though cell culture and transfection experiments also illustrated differences between the two receptors at cellular and molecular levels [66]. In a recent review, the authors concluded that it is reasonable to assume that CXCR2 directly activates PMNs whereas CXCR1 may be able to slow down migration by down-regulation of CXCR2 and acts more in activating microbicidal properties [66].

Gernez et al. summarized that CXCR2 inhibitors are likely to affect all four pools of neutrophils [67]. These pools are: (1) bone marrow reservoir; (2) circulating pool; (3) neutrophils adherent to the endothelial layer; (4) tissue pool.

Generally, G protein blockers block only around 50% of activity of the corresponding receptor. Our group showed that heterozygote mice for CXCR2 still had a significant reduction on PMN migration in the interstitium of the lung and alveolar space [48] and therefore blocking 50% of the receptor is still effective. Results with different pharmacologic agents blocking CXCR2 confirm our results as shown below.

*Reparixin* (formerly known as repertaxin), a non-competitive allosteric inhibitor of CXCR1 and CXCR2 with a 400-fold higher efficacy in inhibiting CXCR1 activity than CXCR2, has been described [35]. Reparixin leads to the modulation of PMN recruitment and therefore tissue damage in experimental models of ischemia/reperfusion injury [35–37] and organ transplantation [68]. Prophylactic and therapeutic application of Reparixin reduced PMN trafficking, vascular permeability, and improved gas exchange in a murine model of LPS-/acid-induced ALI [34] (Table 2). Currently, clinical development is in process.

A potent dual inhibitor of CXCR1 and CXCR2, *DF 2156A,* has recently been investigated [39]. The pharmacologic agent blocks the signal transduction leading to chemotaxis as a non-competitive inhibitor. In a murine model of induced angiogenesis, DF 2156A attenuated infiltration of leukocytes, TNF-*α* production and neovessel formation [39]. DF 2156A also decreased monocyte and PMN infiltration in a model of rat liver ischaemia and reperfusion injury [39]. *In vitro*, the pharmacologic agent attenuated proliferation, migration and capillary-like organization of human umbilical vein endothelial cells after stimulation with human IL-8 [39]. Bertini and colleagues characterized DF 2156A as an effective inhibitor of CXCR1/2-mediated injury in acute and chronic inflammation [39] (Table 3).

DF 2156A was also analyzed in a rat model of ischemia/reperfusion induced by middle cerebral artery occlusion and led to a decrease in PMN infiltration, infarct size and significantly improved neurological function [40].

ELR-CXC chemokine antagonist *CXCL8(3–74)K11R/G31P* improved the course of LPS-induced lung inflammation in pigs as shown by decreased neutrophil migration into the lung (approximately 86%) and reduced TNF*α* (approximately 70%) and IL-1 (approximately 83%) levels in the BAL [42]. CXCL8(3–74)K11R/G31P was also tested in a pig model of pulmonary aspiration and led to reduced pulmonary neutrophil response with significantly reduced pathology but still efficient pulmonary bacterial clearance [41]. Additionally, this specific antagonist reduced radiation-induced alveolitis, but not fibrosis [28].

*Sch527123*, a potent CXCR1 and CXCR2 antagonist with higher affinity to CXCR2, was found to be effective in LPS-induced ALI to reduce pulmonary PMN migration and goblet cell hyperplasia in mice, rats and monkeys [43]. The authors consider this specific antagonist as a possible treatment of inflammatory lung disorders in which pulmonary neutrophilia and mucus hypersecretion are the leading symptoms, for example COPD (chronic obstructive pulmonary disease), asthma, cystic fibrosis, and ARDS. Sch527123 is also a potent, selective antagonist of the human CXCR1 and CXCR2, as shown in *in vitro* assays [45]. Sch527123 is also effective when given orally. In healthy humans, SCH527123 inhibited ozone-induced neutrophil recruitment [46]. The authors concluded that further evaluation in a large trial of patients with pulmonary disorders is warranted.

A recently published study investigated the effect of *simvastatin* in streptococcal-induced ALI [44]. In a murine model, simvastatin led to a decrease of PMN infiltration, most likely through reduced pulmonary release of CXC chemokines.

GSK1325756, a potent, competitive CXCR2 receptor antagonist developed for the treatment of chronic obstructive pulmonary disease (COPD) is currently investigated with the question how the pharmacokinetic is affected by age, gender and food (high fat meal) (http://www.clinicaltrials.gov/). Currently, there is no data available on the effects of this drug in ALI.

*SB-656933*, a selective CXCR2 antagonist, was investigated in ozone-induced airway inflammation in healthy humans [47]. SB-656933 revealed dose-dependent effects on neutrophil activation and recruitment within a well-tolerated dose range. The authors concluded that this agent may be effective in neutrophil-predominant diseases. Currently, one clinical study on the safety, tolerability, steady state pharmacokinetics and pharmacodynamics of SB-656933 in repeated doses is carried out (http://www.clinicaltrials.gov/).

## 6. Blocking CXCR2—Think Twice

In certain inflammatory diseases with special pathogens, a functional CXCR2 is required for neutrophil recruitment and pathogen elimination. CXCR2 knockout mice or antagonism of the receptor led to massive bacterial/fungal outgrowth in *Pseudomonas aeruginosa *[69], *Nocardia asteroides *[70], *Aspergillus fumigatus* [71], and *Streptococcus pneumonia* [72]—infections.

Therefore, further studies on blocking CXCR2 under different disease conditions are needed to understand its mode of action and to find out interactions with other receptors. This attempt is quite difficult since typically, upon activation of ligands, chemokine receptors are quickly desensitized and internalized and therefore have only around 30 seconds for signal transmitting [13].

However, there is one recently discovered example of formerly unknown interactions: receptor oligomerization can lead to a novel signal cascade besides the normal signal pattern. One group found a hetero-oligomerization of the human DOP opioid and CXCR2 receptors [73]. Thereby, a CXCR2 antagonist increased the function of DOP receptor agonists, but only if CXCR2 was expressed. The study suggests that a CXCR2 receptor antagonist is capable to enhance a DOP receptor agonist by acting as an allosteric regulator of a receptor which is a heterodimer partner for the CXCR2 receptor [73].

## 7. Conclusion

In summery, CXCR2 plays a pivotal role in ALI with regard to neutrophil recruitment from the bone marrow, the circulating pool, neutrophils adherent to the endothelium, and the tissue pool. Pharmacologic blocking strategies appear promising in many disease conditions, and in several experimental studies blocking CXCR2 has been shown to attenuate tissue damage und disease progress. Pharmacologic blocking agents are even orally available and offer promising results in humans, but further studies with a larger cohort are needed. There are further investigations on this receptor necessary about its functions and interactions in relation to other receptors.

## Figures and Tables

**Table 1:** CXCR2 ligands in mice and humans.

Systematic name	Receptor	Mouse	Human
CXCL1	CXCR2	KC	GRO*α*, MGSA
CXCL2	CXCR2	MIP-2	GRO*β*, MIP-2*α*
CXCL3	CXCR2	MIP-2	GRO*γ*, MIP-2*β*
CXCL5	CXCR2	LIX	ENA-78
CXCL6	CXCR1,2	CK*α*-3	GCP-2
CXCL7	CXCR2	n/a	NAP-2
CXCL8	CXCR1,2	n/a	IL-8

CXCR1, 2 binding ligands modified from ref. [33]. GRO: growth-related oncogene; MGSA: melanoma growth stimulatory activity; KC: keratinocyte-derived chemokine; MIP: macrophage inflammatory protein; PF: platelet factor; ENA: epithelial cell-derived neutrophil-activating factor; LIX: lipopolisaccharide-induced CXC human chemokine; GCP: granulocyte chemotactic protein; CK: chemokine; NAP: neutrophil-activating protein; IL: interleukin.

**Table 2:** Pharmacological approaches on CXCR2 in animals.

Intervention	Model	Results	Reference
Blocking CXCR2 by a self-made antagonist*	Lung transplant ischemia-reperfusion injury in rats	(i) Marked reduction of neutrophils into the lung (ii) Attenuated graft injury	[18]
Blocking CXCR2 by a self-made antagonist*	Ventilator-induced ALI in mice	(i) Reduced PMN sequestration and ALI	[17]
Reparixin	LPS-/acid-induced ALI in mice	(i) Modulation of PMN trafficking (ii) Reduced vascular permeability (iii) Improved gas exchange	[34]
Reparixin	Reperfusion injury in rats and human/rodent PMNs	(i) Effective inhibitor of PMN recruitment (ii) Attenuated reperfusion injury	[35]
Reparixin	Cerebral ischemia in rats	(i) Anti-inflammatory and neuroprotective effects (ii) Inhibition of PMN infiltration (iii) Reduced tissue damage	[36]
Reparixin	Intestinal ischemia and reperfusion injury in rats	(i) Reduced neutrophil influx (ii) Reduced microvascular permeability in lungs and intestines (iii) Reduced TNF*α* (iv) Reduced lethality	[37]
DF2162	Bleomycin-induced pulmonary inflammation and fibrosis in mice	(i) Improvement of lung pathology (ii) Reduced collagen deposition (iii) Reduced airway PMN migration (iv) Increased PMN in lung parenchyma	[26]
SCH-N	Cigarette smoke-induced lung inflammation in mice	(i) Reduced PMNs in BAL (ii) Reduced perivascular inflammation (iii) Reduced tissue PMNs	[38]
DF 2156A	Human leukocytes, human umbilical vein cells, ischemia/reperfusion injury in rats	(i) Decreased monocyte and PMN infiltration (ii) Reduced TNF*α* (iii) Attenuated proliferation, migration and capillary-like organization of human umbilical vein endothelial cells	[39]
DF2156A	Ischemia/reperfusion injury in rats	(i) Decrease of PMN infiltration (ii) Reduced infarct size (iii) Improved neurological function	[40]
K11R/G31P	Aspiration pneumonia in pigs	(i) Attenuated neutrophil response (ii) Reduced pathology (iii) Still efficient pulmonary bacterial clearance	[41]
K11R/G31P	LPS-induced lung inflammation in pigs	(i) Decreased PMN migration into the lung (ii) Reduced TNF*α* andl IL-1 levels in the BAL	[42]
K11R/G31P	Radiation-induced lung injury in mice	(i) Reduced alveolitis but not fibrosis	[28]
Sch527123*	Intranasal/intratracheal LPS-administration in mice, and rats, repeated bronchoscopy and lavage in cynomolgus monkey	(i) Suppressed pulmonary neutrophilia (ii) Reduced goblet cell hyperplasia	[43]
Simvastatin	Streptococcal-induced ALI in mice	(i) Reduced infiltration of neutrophils and edema in the lung	[44]

*Selective for CXCR2; the other pharmacologic agents also inhibit CXCR1.

**Table 3:** Pharmacological approaches on CXCR2 in humans.

Intervention	Model	Results	Reference
DF 2156A	Human leukocytes, human umbilical vein cells, ischemia/reperfusion injury in rats	(i) Decreased monocyte and PMN infiltration (ii) Reduced TNF*α* (iii) Attenuated proliferation, migration and capillary-like organization of human umbilical vein endothelial cells	[39]
Sch527123	Human neutrophils	(i) Inhibited neutrophil chemotaxis (ii) Inhibited myeloperoxidase release	[45]
Sch527123	Ozone-induced neutrophil recruitment in humans	(i) Lower sputum neutrophil count	[46]
SB-65933*	Ozone-induced airway inflammation in humans	(i) Lower neutrophil activation and recruitment	[47]

*Selective for CXCR2, the other pharmacologic agents also inhibit CXCR1.
